# Freiburg Neuropathology Case Conference: Contrast-enhancing Brain Lesion 6 Months after Resection and Combined Radiotherapy and Chemotherapy of an Unmethylated but IDH R132H-mutated Glioblastoma Multiforme

**DOI:** 10.1007/s00062-021-01006-4

**Published:** 2021-02-24

**Authors:** T. Demerath, D. Erny, O. Schnell, H. Urbach, M. Prinz, C. A. Taschner

**Affiliations:** 1 Departments of Neuroradiology, Medical Centre, University of Freiburg, Breisacherstraße 64, 79106 Freiburg, Germany; 2 Neuropathology, Medical Centre, University of Freiburg, Freiburg, Germany; 3 Neurosurgery, Medical Centre, University of Freiburg, Freiburg, Germany

**Keywords:** Tumor progression, Pseudoprogression, Radionecrosis, Cerebral abscess, Cerebritis

## Case Report

**Fig. 1:**
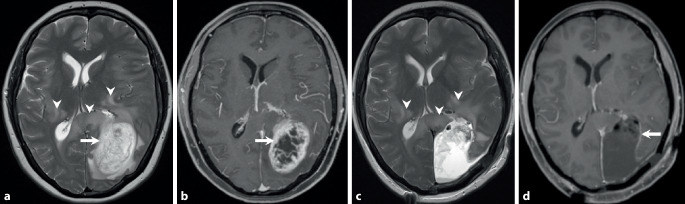
time (t) = 0. Preoperative axial T2-weighted images (**a**) show a hyperintense space-occupying lesion (*arrow*) located in the left occipital lobe. In addition, hyperintense changes are displayed laterally to the thalami of both sides and the occipital forceps of the corpus callosum (**a**, *arrowheads*). After administration of gadolinium (**b**) the lesion shows marked rim enhancement (*arrow*) and central necrosis on T1-weighted images. On postoperative T2-weighted images a large resection defect is visible (**c**). The hyperintense signal intensity changes remain stable *(arrowheads*). On T1-weighted postcontrast images (**d**) at the same point in time complete tumor resection is shown (*arrow*)

**Fig. 2:**
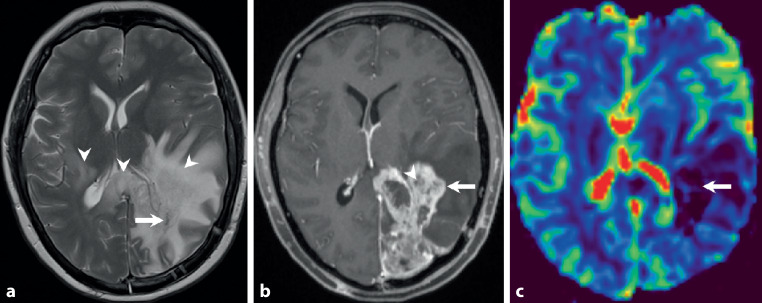
t = 7 weeks. On-going combined radiotherapy and chemotherapy. The hyperintense signal intensity changes have markedly increased on axial T2-weighted images (**a**, *arrowheads*). No signs of a solid residual tumor are on display (**a**, *arrow*). On contrast-enhanced T1-weighted images (**b**) a thick patchy rim of contrast enhancement along the margins of the tumor resection is visible (*arrow*) interleaved with smaller areas of regressive changes (*arrowhead*). On axial cerebral blood volume (CBV) maps of perfusion-weighted MR images (**c**) no hyperperfusion is visible in the areas of increased contrast enhancement (*arrow*)

**Fig. 3:**
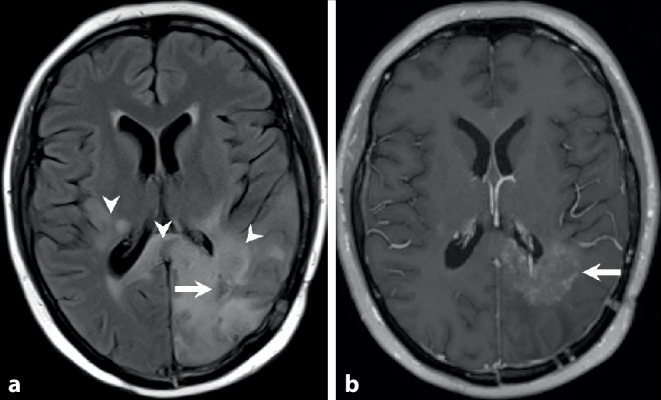
t = 14 weeks. Additional treatment with corticosteroids. The perifocal edema has clearly regressed on axial fluid attenuated inversion recovery (FLAIR) images (**a**, *arrow*). The signal intensity changes of the occipital forceps and the right sided perithalamic region have remained stable (**a**, *arrowheads*). On T1-weighted postcontrast images contrast (**b**) enhancement at the margins of the tumor resection has clearly regressed (*arrow*)

**Fig. 4:**
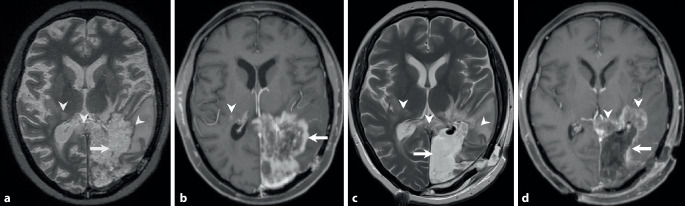
t = 26 weeks. At the end of the second cycle of the intensified chemotherapy with temozolomide the patient complained of mild right-sided hemiparesis. Axial T2-weighted images (**a**) show an increasing perifocal edema (*arrowheads*) around the tumor resection (*arrow*). On axial T1-weighted images after administration of gadolinium (**b**) a thick patchy rim of enhancement can be seen along the margins of the initial tumor resection (*arrow*). Note the additional contrast-enhancing lesion located in the white matter adjacent to the right thalamus (*arrowhead*) most likely corresponding to additional manifestation of the underlying tumor. After reoperation a large resection defect can be seen on axial T2-weighted images (**c**, *arrow*). The perifocal edema is basically stable (**c**, *arrowheads*). On axial contrast-enhanced T1-weighted images (**d**) discrete enhancement is visualized along the borders of the tumor resection (*arrow*). Note the bulky contrast-enhancing portions infiltrating the occipital forceps of the corpus callosum and the white matter lateral to the left thalamus (*arrowheads*)

A 46-year-old woman complained of visual and gait disturbances and increasing headaches that had started some days earlier. Physical examination revealed a hemianopia to the right and restricted orientation to time without additional neurological deficits. A first magnetic resonance imaging (MRI) was performed, which revealed a large contrast-enhancing lesion within the left occipital lobe (Fig. 1). The patient was transferred to the department of neurosurgery at our university hospital. After interdisciplinary discussion in the neuro-oncological board, tumor resection was indicated and performed by use of intraoperative neuronavigation without any additional deficits. Neuropathological examination confirmed an unmethylated but isocitrate dehydrogenase 1 (IDH1) R132H-mutated glioblastoma with a high proliferation rate (MIB‑1: 15–20% immunopositive tumor cells). Combined radiotherapy and chemotherapy with adjuvant temozolomide (TMZ) (EORTC/-NCIC trial protocol [1]) was indicated according to our institutional guidelines. Subsequently, the patient developed steroid-induced psychosis and tapering of steroids along with pentoxifylline and boswellia treatment was recommended. At this time MRI showed significant contrast-enhancement and perilesional edema and dexamethasone was prescribed (Fig. 2). The patient recovered clinically and follow-up MRI displayed partial recovery of brain edema (Fig. 3). After discontinuation of steroids, the patient redeveloped symptoms of depression and MRI showed recurrence of brain edema. At this stage steroids were readministered while continuing with the intensified TMZ chemotherapy. After completion of the second cycle, the patient complained of mild hemiparesis graded as 4/5 on the medical research council (MRC) scale for muscle strength, hemiparesthesia, and constant fatigue. At this point in time MRI showed persisting signs of edema and contrast-enhancement suspicious of tumor progression (Fig. 4).

The indication for reoperation was established at the weekly interdisciplinary neuro-oncological board meeting. Intraoperatively, abundant tissue necrosis was detectable most likely resembling radiation necrosis; however, temporomesially above the tentorium signs of vital tumor were also detectable. Surgery again went well without postoperative deficit. The patient had clinically stable disease for 6 months, before MRI revealed signs of progressive disease. The patient quickly deteriorated clinically and finally succumbed to the disease 16 months after first symptoms had occurred.

## Imaging

On preoperative axial T2-weighted images (Fig. 1a) a hyperintense space-occupying lesion (arrow) located in the left occipital lobe is found. In addition, hyperintense changes are displayed laterally to the thalami of both sides and the occipital forceps of the corpus callosum (Fig. 1a, arrowheads). After administration of gadolinium the lesion shows marked rim enhancement (Fig. 1b, arrow) and central necrosis on T1-weighted images. On postoperative T2-weighted images a large resection defect is visible. The hyperintense signal intensity changes remain stable (Fig. 1c, arrowheads). On T1-weighted postcontrast images at the same point in time complete tumor resection is shown (Fig. 1d, arrow). At 7 weeks after tumor resection with on-going combined radiotherapy and chemotherapy the hyperintense signal intensity changes have markedly increased on axial T2-weighted images (Fig. 2a, arrowhead). No signs of a solid residual tumor are on display (Fig. 2a, arrow). On contrast-enhanced T1-weighted images a thick patchy rim of contrast enhancement along the margins of the tumor resection are visible (Fig. 2b, arrow) interleaved with smaller areas of regressive changes (Fig. 2b, arrowhead). On axial cerebral blood volume (CBV) maps of perfusion-weighted MR images (Fig. 2c) no hyperperfusion is visible in the areas of increased contrast enhancement (Fig. 2c, arrow). At 14 weeks after initial brain tumor surgery under additional treatment with corticosteroids the perifocal edema has clearly regressed on axial FLAIR images (Fig. 3a, arrow). The signal intensity changes of the occipital forceps and the right sided perithalamic region have remained stable (Fig. 3a, arrowheads). On T1-weighted postcontrast images contrast enhancement at the margins of the tumor resection have also markedly regressed (Fig. 3b, arrow). At 26 weeks after initial tumor resection at the end of the second cycle of the intensified chemotherapy with temozolomide the patient complained of mild right-sided hemiparesis. A MRI performed at this time shows increasing perifocal edema on axial T2-weighted images (Fig. 4a, arrowheads) around the tumor resection (Fig. 4a, arrow). On T1-weighted images after administration of gadolinium a thick patchy rim of enhancement can be seen along the margins of the initial tumor resection (Fig. 4b, arrow). Note the additional contrast-enhancing lesion located in the white matter adjacent to the right thalamus (Fig. 4b, arrowhead) most likely corresponding to additional manifestation of the underlying tumor. After reoperation a large resection defect can be seen on axial T2-weighted images (Fig. 4c, arrow). The perifocal edema is basically stable (Fig. 4c, arrowhead). On contrast-enhanced T1-weighted images discrete enhancement is visualized along the borders of the tumor resection (Fig. 4d, arrow). Note the bulky contrast-enhancing portions infiltrating the occipital forceps of the corpus callosum and the white matter lateral to the left thalamus (Fig. 4d, arrowheads).

## Differential Diagnosis

### Tumor Progression

In MRI, tumor progression in glioblastomas usually presents as new or size-progressive contrast uptake showing an increase in relative CBV (rCBV) on dynamic susceptibility contrast (DSC) perfusion imaging. The new lesion can occur at the resection margin as well as distant to the primary tumor area, which is due to the fact that microscopic tumor infiltration into distant brain areas is often already present initially. Less commonly, liquorgenic seeding along the ventricles, basal cisterns, or spinal canal can occur. Tumor progression should ideally be assessed according to the response assessment in neurooncology (RANO) criteria, used in particular in clinical trials to assess standardized follow-up [2]. Progressive disease is defined by an increase in the product of the axial diameters of a measurable lesion of ≥ 25% (compared to baseline) or a significant increase in noncontrast-enhancing areas in T2/FLAIR. In addition, the appearance of any new (enhancing or nonenhancing) lesion components is relevant. The assessment not only requires knowledge about current and previous chemoradiotherapy but also treatment with corticosteroids and the patient’s clinical condition. Perfusion parameters such as rCBV are not included in the RANO assessment but are applied in most centers as an additional tool.

### Pseudoprogression

New or progressive contrast-enhancing areas frequently occur under and after completion of chemoradiotherapy in high-grade but also low-grade gliomas. The RANO criteria define pseudoprogression as new or increasing contrast enhancement that eventually subsides without any change in treatment. Pseudoprogression likely results from increased permeability of the tumor vasculature and radiotherapy-induced inflammation, which may be exacerbated by temozolomide [3]. Histologically, treatment effects such as gliosis and vascular hyalinization and also foci of neoplastic cells often overlap [4].

Pseudoprogression is typically hypointense in T1-weighted and hyperintense in T2/FLAIR sequence with a significant contrast enhancement and optional signs of mass effect. As such it shows the same signal behavior on standard sequences as a real progression. Thus, it is often helpful to additionally perform DSC perfusion, showing usually lower mean rCBV values in pseudoprogression compared to tumor; however, there is a significant overlap and technical factors such as prebolus injection and/or mathematical leakage correction are significant factors that depend not least on the examination protocol and postprocessing being used. Other pitfalls that can interfere with rCBV measurement are marginal hemosiderin deposits, which are often found after tumor resection. In these cases, dynamic contrast enhancement (DCE) perfusion and arterial spin labeling (ASL) may have an advantage, as these are less prone to susceptibility artifacts [5].

### Radionecrosis

The term radionecrosis is often used overlapping with pseudoprogression, but in a narrower sense it refers generally to a late complication of radiation-induced vasculopathy, as a consequence of endothelial cell damage, resulting in obstructive arteriopathy, ischemia, infarction and coagulative necrosis. The prerequisite is that the affected brain area is located in the irradiation field. Radionecrosis also occurs after radiotherapy of, for example, brain metastases or head/neck tumors and also occurs long (12–24 months or even later) after completion of radiotherapy. In MRI radionecrosis is characterized by (potentially space-occupying) contrast-enhancing lesions that also exhibit decreased rCBV, thus overlapping in imaging with the findings of pseudoprogression [6, 7]. Radionecrosis may also be accompanied by T2/FLAIR-hyperintense cerebral edema, predominantly in the cerebral white matter.

### Abscess and Cerebritis

Infectious complications of tumor therapy include intracranial and extracranial abscess formations and concomitant cerebritis of the adjacent brain parenchyma and mostly occur within the subacute postoperative course. Usually, an intra-axial or extra-axial (subdural/epidural) fluid collection is detectable, which typically is hyperintense on T2w sequences showing a homogeneous diffusion restriction with low apparent diffusion coefficient (ADC) values [8]. In typical brain abscesses, a smooth linear rim enhancement is depicted, whereas the enhancement pattern tends to be more inhomogeneous, incomplete and/or “shaggy” in glioblastomas. Furthermore, the dual rim sign, which has been described in susceptibility weighted imaging (SWI) sequences, may be helpful in differentiating between primary abscesses and glioblastomas [9]. Empyemas represent extra-axial collections of pus, more commonly subdurally than extradurally and follow the anatomical boundaries defined by the meninges and similar internal signal as intracerebral abscesses. The adjacent pachymeninges and leptomeninges are often thickened with marked contrast enhancement. Both intracerebral abscesses and intracranial empyema may be accompanied by cerebritis of the adjacent brain parenchyma, characterized by marked vasogenic edema of the brain parenchyma with or without blood-brain barrier disruption.

## Histology

**Fig. 5:**
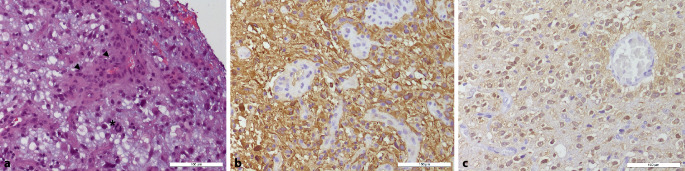
t = 0. Specimen from the initial operation. Hematoxylin-eosin stained section (**a**) showing an astrocytic tumor with endothelial proliferation (*arrowheads*) and mitotic figure (*asterisk*). Size bar: 100 µm. Tumor cells are visualized by immunohistochemical reaction against glial fibrillary acidic protein (GFAP) (**b**). Note the negative reaction within the proliferating endothelial cells. Size bar: 100 µm. Immunohistochemical staining against R132H mutation of IDH1 (**c**) reveals positivity in tumor cells. Size bar: 100 µm

In the hematoxylin-eosin (H&E) stained sections of the formaldehyde-fixed and paraffin-embedded initial biopsy material, a pleomorphic neoplasm with highly increased cellularity with regional signs of necrosis as well as endothelial cell proliferation was observed and classified as glioblastoma multiforme, world health organization (WHO) grade IV. Furthermore, the astrocytic tumor shows predominantly a diffuse growth pattern into the adjacent gliotic central nervous system (CNS) tissue. The tumor cells display mostly small and round-oval shaped chromatin dense nuclei (Fig. 5a). As a sign of increased proliferation, mitotic figures are detectable (Fig. 5a). Staining for GFAP (glial fibrillary acidic protein) is predominantly positive in the tumor tissue (Fig. 5b). Staining using a mutation-specific (R132H) antibody against IDH1, indicative for IDH-mutated astrocytomas and oligodendrogliomas reveals a specific reaction in the present tumor (Fig. 5c).

**Fig. 6:**
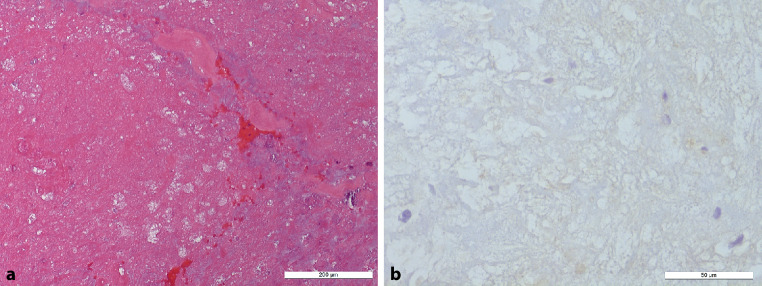
t = 26 weeks*. *Specimen from the reoperation. Hematoxylin-eosin-stained section (**a**) showing necrotic tissue with smaller hemorrhages. Size bar: 200 µm. Immunohistochemical staining against R132H mutation of IDH1 (**b**) shows no detectable tumor cells. Size bar: 50 µm

Bioptic material was received 6 months later from a reoperation with the suspected clinical diagnosis of a recurrent glioblastoma multiforme; however, in the H&E-stained section we observed predominantly CNS tissue with gliosis and larger necrotic areas devoid of viable cells or glial tumor cells as indicated by the absence of basophilic nuclei and prevailing an eosinophilic appearance (Fig. 6a). Regionally, smaller bleedings were present. In addition, IDH1 mutated tumor cells were not detectable (Fig. 6b).

## Diagnosis

### CNS Tissue with Gliosis and Necrosis

In summary, the initial histopathological finding of a glial, pleomorphic, diffuse infiltrating tumor with raised cellularity together with high proliferative activity as well as presence of microvascular proliferation and necrosis leads consequently to the diagnosis of glioblastoma multiforme, WHO grade IV [10]. In contrast, in the second biopsy the histological assessment revealed CNS tissue with gliosis and necrosis, whereby we did not detect a solid glial tumor.

Completeness of resection of enhancing tumor components is associated with prolongation of progression-free survival. In primary (IDH wild type) GBM, > 90% of patients undergo tumor progression within 5 years, which may be accompanied by clinical findings such as progressive or new onset neurologic deficits [11].

Pseudoprogression in glioblastoma is defined as a treatment-related increase in contrast enhancement mimicking tumor progression and classically occurs after combined treatment with chemoradiation, typically involving temozolomide [12, 13]. In total, it occurs in 35–50% of patients, preferably between 3–6 months after conclusion of chemoradiotherapy. Pseudoprogression is more common in O6-methylguanine-DNA methyltransferase(MGMT)-methylated tumors and has been associated with improved survival [14].

In MRI pseudoprogression also tends to have higher ADC-values and lower rCBV than tumor but also may be accompanied by mass effect [15]. To assess (pseudo)progression, knowledge of the patient’s clinical condition and the timing of imaging relative to chemoradiotherapy are essential. In some cases, further follow-up may be necessary to assess (suspected) pseudoprogression, especially if clinical parameters argue against progression or imaging findings are inconclusive. Pseudoprogression may also occur following immunotherapy, is self-limiting and does not require change in treatment [16].

Since in many cases histologic posttherapeutic effects in the tumor area overlap with residual and also progressive microscopic tumor manifestations, and furthermore pseudoprogression often shows similar imaging features of true tumor progression, the findings often require an interdisciplinary decision, which in many cases also requires more specific imaging by e.g. 18F-fluorethyl-l-tyrosine positron emission tomography computed tomography (FET-PET-CT).
